# Enhanced susceptibility to lipopolysaccharide-induced arthritis and endotoxin shock in interleukin-32 alpha transgenic mice through induction of tumor necrosis factor alpha

**DOI:** 10.1186/ar3850

**Published:** 2012-05-21

**Authors:** Masanori Nakayama, Yasuo Niki, Toshiki Kawasaki, Yuki Takeda, Keisuke Horiuchi, Aya Sasaki, Yasunori Okada, Kazuo Umezawa, Hiroyasu Ikegami, Yoshiaki Toyama, Takeshi Miyamoto

**Affiliations:** 1 Department of Orthopaedic Surgery, Keio University, 35 Shinanomachi, Shinjuku, Tokyo 160-8582, Japan; 2 Department of Pathology, Keio University, 35 Shinanomachi, Shinjuku, Tokyo 160-8582, Japan; 3 Department of Applied Chemistry, Faculty of Science and Technology, Keio University, Hiyoshi 3-14-1, Kohoku-ku, Yokohama 223-8522, Japan

## Abstract

**Introduction:**

The present study assessed the potential functions of interleukin (IL)-32α on inflammatory arthritis and endotoxin shock models using IL-32α transgenic (Tg) mice. The potential signaling pathway for the IL-32-tumor necrosis factor (TNF)α axis was analyzed *in vitro*.

**Methods:**

IL-32α Tg mice were generated under control of a ubiquitous promoter. Two disease models were used to examine *in vivo *effects of overexpressed IL-32α: Toll-like receptor (TLR) ligand-induced arthritis developed using a single injection of lipopolysaccharide (LPS) or zymosan into the knee joints; and endotoxin shock induced with intraperitoneal injection of LPS and D-galactosamine. TNFα antagonist etanercept was administered simultaneously with LPS in some mice. Using RAW264.7 cells, *in vitro *effects of exogenous IL-32α on TNFα, IL-6 or macrophage inflammatory protein 2 (MIP-2) production were assessed with or without inhibitors for nuclear factor kappa B (NFκB) or mitogen-activated protein kinase (MAPK).

**Results:**

Single injection of LPS, but not zymosan, resulted in development of severe synovitis with substantial articular cartilage degradation in knees of the Tg mice. The expression of TNFα mRNA in inflamed synovia was highly upregulated in the LPS-injected Tg mice. Moreover, the Tg mice were more susceptive to endotoxin-induced lethality than the wild-type control mice 48 hours after LPS challenge; but blockade of TNFα by etanercept protected from endotoxin lethality. In cultured bone marrow cells derived from the Tg mice, overexpressed IL-32α accelerated production of TNFα upon stimulation with LPS. Of note, exogenously added IL-32α alone stimulated RAW264.7 cells to express TNFα, IL-6, and MIP-2 mRNAs. Particularly, IL-32α -induced TNFα, but not IL-6 or MIP-2, was inhibited by dehydroxymethylepoxyquinomicin (DHMEQ) and U0126, which are specific inhibitors of nuclear factor kappa B (NFκB) and extracellular signal regulated kinase1/2 (ERK1/2), respectively.

**Conclusions:**

These results show that IL-32α contributed to the development of inflammatory arthritis and endotoxin lethality. Stimulation of TLR signaling with LPS appeared indispensable for activating the IL-32α-TNFα axis *in vivo*. However, IL-32α alone induced TNFα production in RAW264.7 cells through phosphorylation of inhibitor kappa B (IκB) and ERK1/2 MAPK. Further studies on the potential involvement of IL-32α-TNFα axis will be beneficial in better understanding the pathology of autoimmune-related arthritis and infectious immunity.

## Introduction

Interleukin-32 (IL-32) was originally identified as natural killer (NK) transcript 4, which is induced by IL-18 in NK cells [1]. NK transcript 4 showed cytokine-like characteristics and played a critical role in inflammation and was therefore renamed IL-32. This cytokine is reportedly produced by NK cells, T cells, epithelial cells, monocytes, and fibroblasts after stimulation by IL-2, IL-12, and IL-18 and interferon-gamma [2]. Initially, four isoforms of IL-32 (IL-32α, β, γ, and δ) derived from alternative splicing of a single gene. Among these, IL-32α is the shortest transcript, whereas IL-32 γ is the longest isoform and has the strongest biological activity [2,3]. Two additional isoforms, IL-32ε and ζ, have recently been identified, but these isoforms are not ubiquitously expressed except in T cells [4].

IL-32 has been shown to exhibit properties typical of a proinflammatory cytokine and to drive the induction of other proinflammatory cytokines and chemokines, such as tumor necrosis factor-alpha (TNFα) and IL-1, IL-6, and IL-8. Owing to such proinflammatory properties, IL-32 has been considered to play a key role in the development of various inflammatory diseases, including rheumatoid arthritis (RA), inflammatory bowel disease [5], mycobacterial [6,7] or viral [8-10] infection, chronic obstetric pulmonary disease [11], and pancreatic tumor [12,13]. Although no receptor or analog of IL-32 has yet been identified in mice, human IL-32 reportedly exerts proinflammatory effects as an inducer of TNFα and other inflammatory cytokines in mice both *in vitro *and *in vivo *[2,14-16].

During the last decade, TNFα and IL-6 became widely perceived as substantial therapeutic targets in RA given that the use of either anti-TNFα or anti-IL-6 therapy could successfully control chronic inflammation in RA. As IL-32 is capable of inducing TNFα and IL-6, this cytokine is increasingly becoming a focus as a potential therapeutic target in RA and other inflammatory disorders. Mounting evidence regarding upstream signaling regulators for IL-32 production has been accumulating in the literature [12,17-20]. However, signaling pathways that are downstream of IL-32 and that lead to TNFα production have yet to be fully elucidated. Most investigators advocate the position that IL-32 augments Toll-like receptor (TLR) signaling, and TLR-2, -3, and -4 are associated with the effects of IL-32 signaling, although the detailed mechanisms remain to be clarified. Only a few studies to date have reported the implications of mitogen-activated protein kinase (MAPK) or nuclear factor kappa B (NF-κB) pathways in IL-32 signaling [2,21-23].

The present study generated IL-32α transgenic (Tg) mice that overexpressed human IL-32α under a control of ubiquitous CAG promoter, and it assessed the *in vivo *effects of IL-32α on TLR signaling in the induction of arthritis and endotoxin shock models using the Tg mice. In addition, the potential signaling pathway of the IL-32α-TNFα axis was analyzed *in vitro*.

## Materials and methods

### Reagents

Lipopolysaccharide (LPS) from *Escherichia coli *0111:B4 and zymosan A from *Saccharomyces cerevisiae *were purchased from Sigma-Aldrich (St. Louis, MO, USA), and D-galactosamine was purchased from Wako Pure Chemical Industries (Osaka, Japan). Etanercept was obtained from Wyeth (Tokyo, Japan). Recombinant human IL-32α protein (rIL-32α) was obtained from Takara Bio (Shiga, Japan). IL-32α-specific enzyme-linked immunosorbent assay (ELISA) (Human IL-32α ELISA MAX Deluxe Set) was purchased from BioLegend (San Diego, CA, USA), and TNFα-specific ELISA (Quantikine Mouse TNFα) and anti-IL-32α antibody were purchased from R&D Systems (Minneapolis, MN, USA). All other antibodies were purchased from Cell Signaling Technology Japan (Tokyo, Japan). Dehydroxymethylepoxyquinomicin (DHMEQ) (an inhibitor for NF-κB) was provided as previously described [24,25]. MAPK inhibitors U0126, SB203580, and SP600125 - which are inhibitors for ERK1/2, p38, and JNK, respectively - were purchased from Sigma-Aldrich. DHMEQ and MAPK inhibitors were dissolved in 100% dimethyl sulfoxide (DMSO) at 100 mg/mL and were stored in aliquots at -30°C. Before use in cell culture, they were diluted with the medium to a final DMSO concentration of not more than 0.05%.

### Real-time polymerase chain reaction analysis

Total RNAs were isolated from each sample by RNAiso plus (Takara Bio), and cDNAs were synthesized from total RNAs by using a PrimeScript RT reagent Kit (Takara Bio). Real-time polymerase chain reaction (PCR) was performed by using SYBR Premix ExTaq II (Takara Bio) with a DICE Thermal cycler (Takara Bio) in accordance with the instructions of the manufacturer. Results were normalized to glyceraldhyde-3-phosphate dehydrogenase (GAPDH) as the fold change compared with samples. The primer sequences used in this study are presented in Table 1.

**Table 1:** Primers used in real-time polymerase chain reaction

Primer	Sense	Sequence
Mouse GAPDH	Forward	5'-CTTTGTCAAGCTCATTTCCTGG-3'
	Reverse	5'-TCTTGCTCAGTGTCCTTGC-3'
Human IL-32α	Forward	5'-CCTTGGCTCCTTGAACTTTTG-3'
	Reverse	5'-CTGTCCACGTCCTGATTCTG-3'
Mouse TNFα	Forward	5'-CTTCTGTCTACTGAACTTCGGG-3'
	Reverse	5'-CAGGCTTGTCACTCGAATTTTG-3'
Mouse IL-6	Forward	5'-CAAAGCCAGAGTCCTTCAGAG-3'
	Reverse	5'-GTCCTTAGCCACTCCTTCTG-3'
Mouse MIP-2	Forward	5'-GAAGTCATAGCCACTCTCAAGG-3'
	Reverse	5'-CTTCCGTTGAGGGACAGC-3'

GAPDH, glyceraldhyde-3-phosphate dehydrogenase; IL, interleukin; MIP-2, macrophage inflammatory protein 2; TNFα, tumor necrosis factor-alpha.

### Immunoblot analysis

Mice organs or cell lysates were prepared by using RIPA buffer: 1% Triton X-100, 1% sodium deoxycholate, 0.1% SDS, 150 mM NaCl, 10 mM Tris-HCl, pH 7.5, 5 mM ethylenediaminetetraacetic acid (EDTA), and a protease inhibitor cocktail. An equal volume of protein from each lysate was separated by SDS-PAGE, and separated proteins were transferred to nitrocellulose membranes. After transfer, the membrane was blocked with 5% non-fat dried skim milk for 1 hour at room temperature and then incubated with the primary antibody against the target molecule overnight. Next, the membrane was incubated with horseradish peroxidase-conjugated secondary antibody for 1 hour at room temperature. After washing, protein was detected by using an enhanced chemiluminescence (ECL) system. The same membrane was then stripped by Tris-HCL (62.5 mM), 2% SDS, and β-mercaptoethanol and blocked with skim milk and incubated with primary antibody against another molecule, following the same procedure mentioned above.

### Generation of human IL-32α transgenic mice

We generated IL-32α Tg mice, which overexpressed human IL-32α under a control of a ubiquitous CAG promoter constructed by the first intron of the chicken β-actin gene and a portion of the rabbit β-globin gene [26]. The background of these mice was C57BL/6 Jcl (obtained from CLEA Japan, Tokyo, Japan). Wild-type C57BL/6 Jcl (Wt) mice were also obtained from CLEA Japan. All mice were 18 to 20 weeks old when they were used. All animal experiments were conducted in accordance with institutional and national guidelines. IL-32α insertion was confirmed by amplification of the genome DNA isolated from mouse organs by using real-time PCR. The reason real-time PCR was employed was that we wanted to simultaneously detect and quantify transgene-derived IL-32α in multiple organs of Tg mice. The amount of TNFα expression in multiple organs was measured by real-time PCR. IL-32α protein in multiple organs was detected by immunoblot analysis. Levels of IL-32α in blood serum, knee, and liver lysate were measured by specific ELISA.

### Inflammatory arthritis model

Inflammatory arthritis was induced as described previously [27-29]. As in these reports, intra-articular injection of LPS was employed in IL-32α Tg mice to elucidate the arthritogenic capacity of IL-32α. Knees of Tg and Wt mice were injected once with LPS (1 μg, much less than the reported amount [27,28]) or zymosan (20 μg, much less than the reported amount [29]) without any other material as a booster. Injection of phosphate-buffered saline (PBS) served as a control to the contralateral knee of the same mouse. Two weeks after injection, a histopathological examination was performed, and TNFα mRNA expression in synovia of knees was quantitatively measured by using real-time PCR.

### Endotoxin shock model

Endotoxin shock was induced by intraperitoneal injections of 5 μg of LPS and 10 mg of D-galactosamine [30]. Half of the mice were injected with 100 μg of etanercept at the same time. All injected mice were closely monitored every hour for the first 16 hours and every 3 to 6 hours thereafter for 48 hours. In an additional experiment, we sacrificed mice at 1, 3, and 6 hours after intraperitoneal injection and extracted the liver, spleen, and blood serum. TNFα mRNA of liver and spleen lysates was measured by real-time PCR, and serum TNFα was measured by ELISA.

### Cell culture

Bone marrow (BM) was extracted from the leg bones of Tg or Wt mice and cultured for 72 hours in alpha-minimum essential medium (Sigma-Aldrich) containing 10% fetal calf serum (FCS), 100 U/mL penicillin, 100 mg/mL streptomycin, and 1% Gluta Max (Gibco Invitrogen, Carlsbad, CA, USA) supplemented with macrophage colony-stimulating factor (50 ng/mL; Kyowa Hakko Kirin, Tokyo, Japan). RAW 264.7 cells were cultured in Dulbecco's modified Eagle's medium (high-glucose) (Gibco Invitrogen) containing 10% FCS, 100 U/mL penicillin, and 100 μg/mL streptomycin.

### Stimulation of cells

BM cells from Tg and Wt mice (10^6 ^cells) were incubated under stimulation of LPS (50 ng/mL) for 1, 3, 6, and 24 hours, and the amount of TNFα was measured by real-time PCR. TNFα concentration in culture media of BM cells (10^5 ^cells) after 24-hour incubation with LPS (5, 10, or 50 ng/mL) was measured by specific ELISA. RAW 264.7 cells (2 × 10^4 ^cells) were cultured with rIL-32α or LPS for 24 hours, and the TNFα concentration in culture media was measured by ELISA. For the analysis of signaling pathway, RAW 264.7 cells (2 × 10^4 ^cells) were stimulated with rIL-32α in combination with the specific inhibitors of NF-κB and MAPKs, such as DHMEQ (0.4, 2, or 10 μg/mL), U0126 (0.2, 1, or 5 μM), SB203580 (0.2, 1, or 5 μM), and SP600125 (0.4, 2, or 10 μM). DMSO (final concentration of 0.05%) served as a control. After 24 hours of culture, the level of TNFα in culture media was measured by ELISA. For immunoblot analysis, RAW 264.7 cells (2 × 10^6 ^cells) were cultured with rIL-32α for 5, 10, 30, 60, 90, 120, or 180 minutes, followed by washing with ice-cold PBS and lysis in RIPA detergent buffer. The resultant cell lysates were then immunoblotted by using affinity-purified antibodies against phospho-IκB, IκB, phospho-ERK1/2, ERK1/2, phospho-p38, p38, phospho-JNK, JNK, and β-actin. mRNA expressions for IL-6 and macrophage inflammatory protein 2 (MIP-2) as well as TNFα in IL-32-stimulated RAW 264.7 cells (2 × 10^4 ^cells) were measured by real-time PCR, and the inhibitory effects of specific signaling inhibitors, including DHMEQ, U0126, SB203580, and SP600125, were analyzed.

### Statistical analysis

Results are reported as the mean ± standard deviation. Statistical analysis was undertaken by using a two-tailed Student *t *test. Differences were considered statistically significant at a *P *value of less than 0.05. The endotoxin shock model was graphed in Kaplan-Meier format and analyzed by a log-rank test. All experiments were performed in three or four times.

## Results

### Generation of human IL-32α transgenic mice

**Figure 1:**
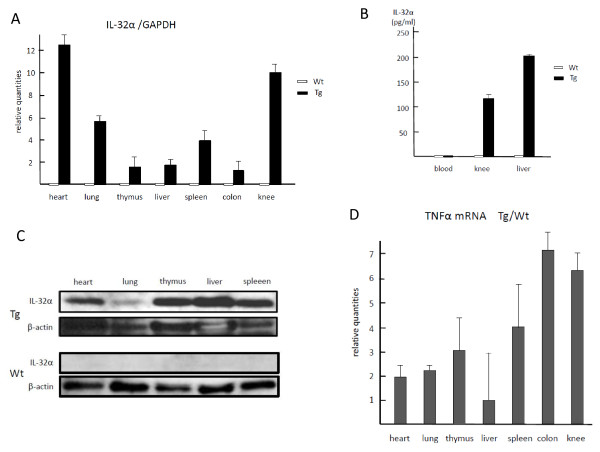
**Overexpression of transgene-derived interleukin-32 alpha (IL-32α) in transgenic (Tg) mice**. Levels of transgene-derived human IL-32α mRNA in various organs were measured by real-time polymerase chain reaction. IL-32α mRNA expression was prominent in knee joints and heart **(a)**. Transgene-derived IL-32α protein was not detected in serum from Tg mice **(b) **but was detected in multiple organs **(c)**. Levels of tumor necrosis factor-alpha (TNFα) mRNA constitutively expressed in various organs were analyzed for Tg and wild-type (Wt) mice. The relative quantities of Tg were divided by those of Wt of each organ and that ratio is graphed **(d)**. In particular, TNFα expression levels in colon and knee joint of Tg mice corresponded to six- to seven-fold those of their littermates. The values are expressed as the mean ± standard deviation for four different mice. GAPDH, glyceraldhyde-3-phosphate dehydrogenase.

Human IL-32α Tg mice were designed to overexpress human IL-32α by using CAG promoter. Of seven F0 mice, two mice expressing sufficient levels of IL-32α mRNA were used to establish lines. The F0 mice and all offspring exhibited no evident pathological phenotype, had a normal body weight, and developed and bred normally. Real-time PCR analysis of the Tg mouse lines demonstrated high levels of IL-32α mRNA expression in a variety of organs, prominently in the knee joint and cardiac muscle (Figure 1a). Transgene-derived IL-32α protein could be detected in multiple organs (Figure 1b, c) but not in serum from Tg mice (Figure 1b). This result might be because the IL-32α isotype has been reported to remain intracellularly [2]. Constitutive expression of TNFα mRNA induced by overexpressed IL-32α was apparent in most organs, and expression levels in the colon and knee joint from Tg mice reached six to seven times the levels seen in littermates (Figure 1d).

### Single intra-articular injection of LPS, but not zymosan, induced inflammatory synovitis and cartilage degradation in transgenic mice

**Figure 2:**
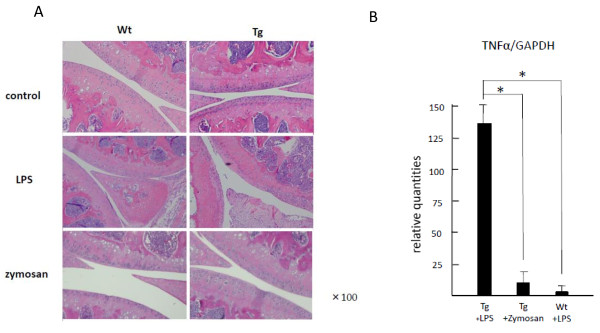
**Inflammatory arthritis induced by single intra-articular injection of lipopolysaccharide (LPS) in transgenic (Tg) mice**. One microgram of LPS or 20 μg of zymosan was injected into the knee joints of Tg and wild-type (Wt) mice. Histopathologically, severe synovitis with articular cartilage destruction was observed 2 weeks after injection in Tg mice injected with LPS but not in Tg mice injected with zymosan or phosphate-buffered saline or in Wt mice (×100) **(a)**. The level of tumor necrosis factor-alpha (TNFα) mRNA expressed in inflamed synovia was significantly higher in Tg mice injected with LPS than in Tg mice injected with zymosan or in Wt mice injected with LPS (*P < 0.01) **(b)**. The values are expressed as the mean ± standard deviation for four different mice. GAPDH, glyceraldhyde-3-phosphate dehydrogenase.

Mice were sacrificed 2 weeks after a single injection of LPS or zymosan, followed by a histopathological examination of the knee joints. The results indicated that the single injection of LPS, but not zymosan, resulted in the development of severe synovitis with articular cartilage destruction in the knees of Tg mice. Such LPS-induced arthritis did not occur in the knees of Wt mice or in contralateral knees injected with PBS (Figure 2a). The level of TNFα mRNA expressed in inflamed synovia after LPS injection was significantly higher in Tg mice than in Wt mice (Figure 2b).

### Transgenic mice exhibited severe endotoxin lethality after LPS challenge

**Figure 3:**
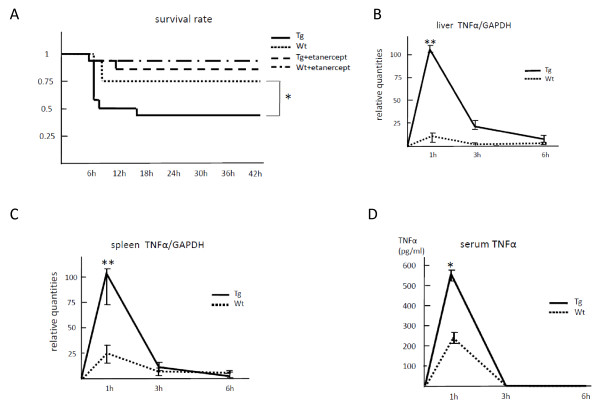
**Interleukin-32 alpha overexpression in mice increased the lethality of lipopolysaccharide (LPS)-induced endotoxin shock**. Endotoxin shock was induced in both transgenic (Tg) mice (*n *= 24) and wild-type (Wt) mice (*n *= 24) by intraperitoneal injection of LPS with D-galactosamine. Twelve mice of each group were simultaneously administered etanercept. All mice were monitored for survival up to 48 hours. Survival rate was significantly worse in the Tg group than in the Wt group (*P < 0.05). Etanercept protected against endotoxin shock in both groups (a). Temporal changes in TNFα levels were measured by real-time polymerase chain reaction for liver and spleen lysate **or**. enzyme-linked immunosorbent assay for blood serum. Levels of tumor necrosis factor-alpha (TNFα) mRNA and protein peaked at 1 hour after LPS injection in the liver (b), spleen (c), and serum (d) from Tg and Wt mice, but levels were much higher in Tg mice than in Wt mice (*P < 0.05, **P < 0.01). The values are expressed as the mean ± standard deviation for four different mice. GAPDH, glyceraldhyde-3-phosphate dehydrogenase.

As a single intraperitoneal injection of LPS with D-galactosamine has been perceived to be capable of inducing endotoxin shock in mice, the impacts of constitutive expression of IL-32α and subsequently produced TNFα on endotoxin lethality were investigated. Mice receiving an intraperitoneal injection of LPS started to die in 5 hours, and the survival rates at 48 hours after injection were 41% for Tg mice and 75% for Wt mice (Figure 3a), showing statistical significance (*P *< 0.05). Importantly, blockade of TNFα by simultaneous administration of etanercept protected from endotoxin shock and markedly increased survival rate in both Tg and Wt mice, suggesting that IL-32-induced TNFα played a key role in developing endotoxin shock. TNFα mRNA expression in liver and spleen peaked at 1 hour after injection and at much higher levels than those of Wt mice (*P *< 0.01) (Figure 3b, c). A similar temporal course was observed for the level of TNFα protein in serum (Figure 3d). At 1 hour after injection, levels of TNFα mRNA in the liver and spleen and TNFα protein in serum were significantly higher in Tg mice than in Wt mice (*P *< 0.05).

### Endogenously overexpressed IL-32α accelerated production of TNFα upon stimulation with LPS

**Figure 4:**
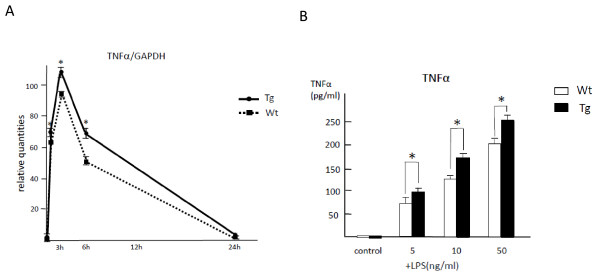
**Bone marrow (BM) macrophages from transgenic (Tg) mice were stimulated to produce tumor necrosis factor-alpha (TNFα) upon stimulation with lipopolysaccharide (LPS)**. Temporal changes in the level of TNFα mRNA expressed by BM macrophages were measured upon stimulation with LPS. TNFα mRNA level peaked at 3 hours and gradually decreased and was significantly higher in Tg mice than in wild-type (Wt) mice over time **(a)**. The amount of TNFα in culture media was measured after 24 hours of LPS stimulation by using a specific enzyme-linked immunosorbent assay **(b)**. LPS stimulated secretion of TNFα from BM macrophages in a dose-dependent manner, and levels were higher in Tg mice than in Wt mice (*P < 0.05). The values are expressed as the mean ± standard deviation for three different mice. GAPDH, glyceraldhyde-3-phosphate dehydrogenase.

To examine the effects of endogenous IL-32α on TNFα production *in vitro*, BM macrophages derived from IL-32α Tg and Wt mice were used. The level of TNFα mRNA expression was significantly higher in Tg mice than in Wt mice after stimulation with LPS (*P *< 0.05). Temporal changes in TNFα mRNA expression revealed that the level of TNFα mRNA peaked at 3 hours after LPS stimulation and gradually decreased with time (Figure 4a). LPS increased TNFα secretion into culture media in a dose-dependent manner, and the amounts of TNFα produced by BM macrophages were universally higher in Tg mice than in Wt mice throughout all doses of LPS tested (Figure 4b).

### Exogenous IL-32α enhanced TNFα production in RAW 264.7 cells through NF-κB and ERK1/2 signaling pathways

**Figure 5:**
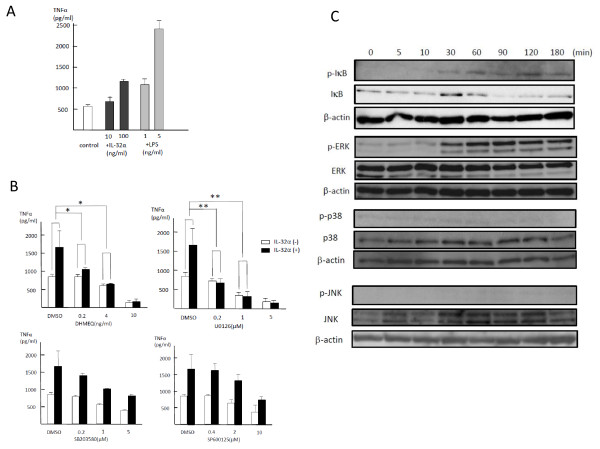
**Exogenous 'IL-32α induced' TNFα production by RAW 264.7 cells through activation of NF-κB and ERK1/2 MAPK**. Two different concentrations of rIL-32α or LPS were added to RAW 264.7 cells in culture, followed by incubation for 24 hours. The level of TNFα in culture media was measured by a specific enzyme-linked immunosorbent assay **(a)**. IL-32α alone as well as LPS was capable of inducing TNFα secretion into culture media by RAW 264.7 cells. Values are expressed as ± standard deviation (SD) of four determinations. RAW 264.7 cells were cultured with or without rIL-32α for 24 hours in combination with inhibitors for IκB and MAPKs, including DHMEQ, U0126, SB203580, and SP600125 **(b)**. IL-32α-induced TNFα production was inhibited by DHMEQ or U0126 but not by SB203580 or SP600125 (*P < 0.05, **P < 0.01). Values are expressed as ± SD of four determinations. Phosphorylations of IκB and MAPKs in RAW 264.7 cells were determined by Western blotting by using anti-phospho-IκB, -ERK1/2, -p38, or -JNK antibodies after treatment with rIL-32α (100 ng/mL) **(c)**. rIL-32α stimulated phosphorylation of IκB and ERK1/2, starting at 30 minutes and peaking at 90 (ERK1/2) or 120 (IκB) minutes, whereas significant phosphorylation was not observed in p38 or JNK. Degradation of IκB was also observed with a peak at 90 minutes. These data represent one of three independent experiments. DHMEQ, dehydroxymethylepoxyquinomicin; DMSO, dimethyl sulfoxide; IκB, inhibitor kappa B; IL, interleukin; LPS, lipopolysaccharide; MAPK, mitogen-activated protein kinase; NF-κB, nuclear factor kappa B; rIL-32α, recombinant human interleukin-32α protein; TNFα, tumor necrosis factor-alpha.

To elucidate the effects of exogenous IL-32α on TNFα production *in vitro*, rIL-32α was added to RAW 264.7 cells in culture. Although RAW 264.7 cells constitutively produced substantial amounts of TNFα, rIL-32α alone as well as LPS could further stimulate RAW 264.7 cells to produce TNFα (Figure 5a). DHMEQ and U0126, as inhibitors of NF-κB and ERK1/2, respectively, reduced IL-32α-induced TNFα production in a dose-dependent manner, whereas SB203580 and SP600125, as inhibitors of p38 and JNK, respectively, did not (Figure 5b). Immunoblot analysis revealed that exogenous IL-32α clearly phosphorylated IκB and ERK1/2, both starting at 30 minutes and peaking at 90 minutes for ERK1/2 and at 120 minutes for IκB, whereas significant phosphorylation was not observed in p38 or JNK (Figure 5c). These results supported the finding that DHMEQ and U0126, but not SB203580 and SP600125, inhibited IL-32α-induced TNFα production. Consequently, exogenous IL-32α-induced TNFα production was mediated predominantly through the activation of NF-κB and the MEK-ERK signaling pathway.

### Exogenous IL-32α stimulated IL-6 and MIP-2 expression in RAW 264.7 cells independently of NF-κB and MAPK signaling pathways

**Figure 6:**
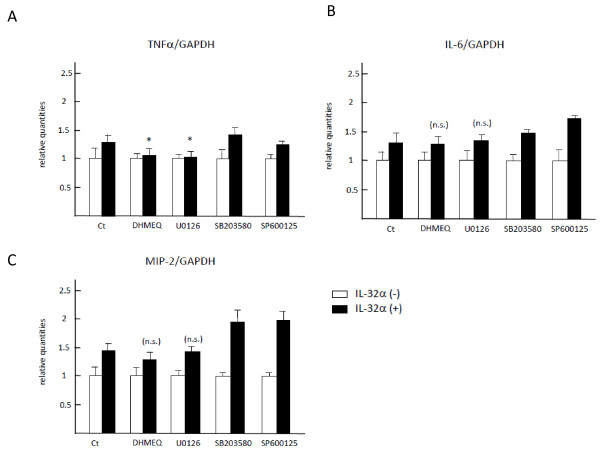
**Exogenous IL-32α stimulated mRNA expression of IL-6 and MIP-2 independently of NF-κB and MAPK signaling pathways**. RAW 264.7 cells were stimulated with rIL-32α in combination with inhibitors for IκB and MAPKs, including DHMEQ, U0126, SB203580, and SP600125, for 6 hours. The amounts of mRNA of TNFα **(a)**, IL-6 **(b)**, and MIP-2 **(c) **were measured by real-time polymerase chain reaction. rIL-32α alone stimulated RAW 264.7 cells to express IL-6 and MIP-2 as well as TNFα at the mRNA level. None of the inhibitors canceled IL-6 and MIP-2 mRNA expression induced by IL-32α, whereas DHMEQ and U0126 suppressed TNFα mRNA expression. Values are expressed as mean ± standard deviation of triplicate determinations. Ct, control; DHMEQ, dehydroxymethylepoxyquinomicin; GAPDH, glyceraldhyde-3-phosphate dehydrogenase; IκB, inhibitor kappa B; IL, interleukin; LPS, lipopolysaccharide; MAPK, mitogen-activated protein kinase; MIP-2, macrophage inflammatory protein 2; NF-κB, nuclear factor kappa B; n.s., not significant; rIL-32α, recombinant human interleukin-32α protein; TNFα, tumor necrosis factor-alpha.

The effects of exogenous IL-32α on IL-6 and MIP-2 production were examined since these cytokines were reportedly induced by IL-32 [3]. rIL-32α alone stimulated RAW 264.7 cells to express TNFα, IL-6, and MIP-2 mRNAs to a similar degree. Specific signaling inhibitors, DHMEQ and U0126, suppressed the expression of TNFα mRNAs; however, neither of these two inhibitors affected the expression of IL-6 and MIP-2 mRNAs induced by IL-32α (Figure 6), suggesting that a signaling pathway other than NF-κB and MAPKs might be involved in IL-6 and MIP-2 mRNA expressions.

## Discussion

To date, the arthritogenic role of IL-32 has been elucidated on the basis of accumulated evidence that overexpression of IL-32β in a mouse model using bone transplantation exacerbated collagen-induced arthritis in mice [14] and that intra-articular injection of IL-32γ in mouse knee joints resulted in severe joint inflammation [15]. In this study, although IL-32α Tg mice did not spontaneously exhibit any abnormal phenotype, intra-articular injection of low-dose LPS resulted in the development of inflammatory arthritis. However, injection of zymosan was not capable of sufficiently inducing TNFα and subsequent arthritis. As LPS is known as a specific ligand of TLR-4, interaction of IL-32α with TLR-4 may play a critical role in the development of arthritis, and this was also the case in LPS-triggered endotoxin shock in the Tg mice. This endotoxin shock model provided an excellent means to evaluate the effects of IL-32α on infectious immunity. In the present study, IL-32α overproduction in Tg mice was associated with severe endotoxin lethality; this was shown to be mediated through the induction of TNFα, because etanercept significantly attenuated the endotoxin shock.

Although the present study clearly demonstrated that LPS, as a TLR-4 agonist, but not the TLR-2 agonist zymosan, might play a key role in potentiating the proinflammatory activity of IL-32α, how exactly IL-32α interacted with the TLR-4 signaling pathway remains unclear. Most recently, Heinhuis and colleagues [31] reported that LPS co-stimulation was mandatory to elicit IL-32 bioactivity in THP-1 cells, and the present study obtained similar findings that TNFα production promoted by IL-32α required co-stimulation with LPS (Figure 4). In terms of the interaction between IL-32 and TLR-2/NOD2 (nucleotide-binding oligomerization domain-containing protein 2) signaling, IL-32 has been reported to stimulate TNFα, IL-6, and IL-8 production by directly increasing expression of TLR-2 and NOD2 [32]. Conversely, the interaction of IL-32 with TLR-4 can be speculated to involve the binding of IL-32 to its putative receptor modulates downstream signaling for TLR-4 or other TLRs, since the proinflammatory activities of IL-32 were present even in macrophages derived from TLR-4^-/- ^mice, and stimulation with IL-32 plus TLR ligand elicited only additive effects rather than synergistic effects [33]. Two candidate molecules potentially connecting IL-32α and TLR-4 signaling are considered. One is proteinase-3 and the other is proteinase-activated receptor 2 (PAR2); the former reportedly acts as an IL-32-binding protein and cleaves all isoforms of IL-32 to generate a more active form [34,35], and the latter has been shown to be associated with late NF-κB activation and subsequent TNFα production predominantly through a myeloid differentiation factor 88 (MyD88)-independent pathway [36].

In contrast to mounting evidence on upstream signaling regulators for IL-32, downstream signaling pathways of IL-32 toward TNFα production have not yet been analyzed in detail, and only a small number of reports have focused on different signals in different cell types. The first report of IL-32 advocated that IL-32α stimulated TNFα production through activation of NF-κB and p38 MAPK in mouse RAW 267.4 cells; the two peaks of p38 MAPK phosphorylation at 5 and 45 minutes were considered a characteristic finding for this cytokine [2]. Netea and colleagues [21] subsequently reported that IL-32-induced TNFα production by human peripheral blood mononuclear cells (PBMCs) was similarly regulated through phosphorylation of p38 MAPK. On the other hand, ERK1/2 MAPK was dominant in IL-32-induced osteoclastogenesis for human PBMCs [22] and in IL-32-induced IL-6 and IL-8 production by human fibroblast-like synoviocytes [23]. The present study demonstrated that IL-32α-induced TNFα production was mediated through phosphorylation of IκB and ERK1/2 in RAW 267.4 cells. Actually, all three components of MAPKs - p38, JNK, and ERK1/2 - were constitutively phosphorylated in RAW 267.4 cells; however, only ERK1/2 phosphorylation was significantly accelerated in response to IL-32α stimulation. This observation corroborated the fact that the addition of inhibitors for ERK1/2 and NF-κB suppressed each phosphorylation (data not shown and Figure S1 of Additional file 1) and consequently canceled IL-32α-induced upregulation of TNFα at the mRNA level (Figures 5b and 6). Given the delayed phosphorylation of IκB and ERK1/2 starting at 30 minutes in this study, IL-32α in RAW 267.4 cells might not directly activate IκB or ERK1/2. Instead, other molecules might play an important role in IκB or ERK1/2 activation of IL-32α, or the IL-32α-TNFα axis might use the MyD88-independent pathway reportedly associated with the late inflammatory response of TLR-4 [37]. Our study also revealed that IL-32α induced IL-6 and MIP-2 as well as TNFα, but their induction was not canceled by inhibitors for NF-κB or MAPKs. This observation indicates that a signaling pathway other than NF-kB or MAPKs might be involved in IL-6 and MIP-2 expressions.

IL-32γ Tg mice obtained by using a promoter similar to that of the present study reportedly exhibited no apparent phenotype, but once inflammatory colitis was induced with dextran sodium sulfate (DSS) in the Tg mice, severe colitis occurred within 4 days [38]. Interestingly, at more than 6 days after DSS challenge, the degree of colonic inflammation in the Tg mice was significantly reduced, and recovery was more rapid than that in Wt mice because of increased IL-10 levels in serum. In another study, IL-32β was reported to promote the production of IL-10 in human cell lines [39]. According to our data on RAW 264.7 cells, the level of TNFα in culture media peaked at 12 hours after stimulation with IL-32α and gradually decreased thereafter, whereas IL-10 levels increased from 24 to 96 hours after stimulation (data not shown and Figure S2 of Additional file 2). IL-32 is thus considered to represent a cytokine possessing contradictory properties according to the different phases of the disease. Such paradoxical effects of IL-32 were not observed in our Tg mice. In fact, a single intra-articular injection of LPS in our Tg mice resulted in a transient flare of inflammatory arthritis, characterized by neutrophil infiltration and synovial proliferation, but such inflammation might cease within 2 weeks, followed by amelioration of synovitis with only mild cartilage erosion remaining. On the other hand, the endotoxin shock model using our Tg mice was suitable for examining short-term effects, but not long-term effects, of IL-32 *in vivo *since most mice died within several hours after LPS challenge, and TNFα induced by IL-32α and LPS was confirmed as an early mediator of endotoxin lethality [40]. The time-dependent and complicated regulation of IL-32 and the relevant molecules of the IL-32-TNFα axis during the course of autoimmune-related arthritis and infectious immunity should be elucidated in future studies.

## Conclusions

This study revealed that IL-32α contributed to the development of LPS-induced inflammatory arthritis and endotoxin lethality; therefore, stimulation of LPS appears indispensable for activating the IL-32-TNFα axis *in vivo*. However, IL-32α alone induced TNFα production in RAW 264.7 cells through phosphorylation of IκB and ERK1/2 MAPK.

## Abbreviations

BM: bone marrow; DHMEQ: dehydroxymethylepoxyquinomicin; DMSO: dimethyl sulfoxide; DSS: dextran sodium sulfate; ELISA: enzyme-linked immunosorbent assay; ERK1/2: extracellular signal regulated kinase1/2; FCS: fetal calf serum; IκB: inhibitor kappa B; IL: interleukin; LPS: lipopolysaccharide; MAPK: mitogen-activated protein kinase; MIP: macrophage inflammatory protein; MyD88: myeloid differentiation factor 88; NF-κB: nuclear factor kappa B; NK: natural killer; NOD: nucleotide-binding oligomerization domain-containing protein; PBMC: peripheral blood mononuclear cell; PBS: phosphate-buffered saline; PCR: polymerase chain reaction; RA: rheumatoid arthritis; rIL-32α: recombinant human interleukin-32α protein; Tg: transgenic; TLR: Toll-like receptor; TNF: tumor necrosis factor; Wt: wild-type (C57BL/6 Jcl).

## Competing interests

The authors declare that they have no competing interests.

## Authors' contributions

MN carried out the animal and molecular experiments, performed the statistical analysis, and drafted the manuscript. YN conceived and designed the study and edited the manuscript. TK taught the experimental procedure and advised MN on this study. YTa and KH were involved in the conception and design of the study. AS and YO performed the histopathological examinations. KU provided the reagents to MN. HI, YTo, and TM helped to supervise the study design and provide valuable advice to MN. All authors read and approved the final manuscript.

## Supplementary Material

Additional file 1 **Figure S1**. Phosphorylation of IκB and MAPKs stimulated with rIL-32α (100 ng/ml) in RAW 264.7 cells was determined by Western blotting using anti-phospho-IκB, -ERK1/2, -p38, and -JNK antibodies. Phosphorylation of IκB and ERK1/2 were observed and inhibited by their specific inhibitors, while significant phosphorylation of p38 or JNK. was not observed. This data represents one of three independent experiments. Click here for file

Additional file 2 **Figure S2**. Levels of TNFα and IL-10 were expressed as a proportion to that in control culture media without IL-32α stimulation. Level of TNFαpeaked at 12 h after stimulation with IL-32α and gradually decreased thereafter, while levels IL-10 kept increasing from 24 to 96 h after stimulation. Values are expressed as mean ± SD of triplicate determinations. Click here for file
