# Supplementary figures and images for: Enhanced susceptibility to lipopolysaccharide-induced arthritis and endotoxin shock in interleukin-32 alpha transgenic mice through induction of tumor necrosis factor alpha
Source: Arthritis Res Ther. 2012 May 21;14(3):R120. doi: 10.1186/ar3850 (PMC3446501; doi:10.1186/ar3850)

Figure S1

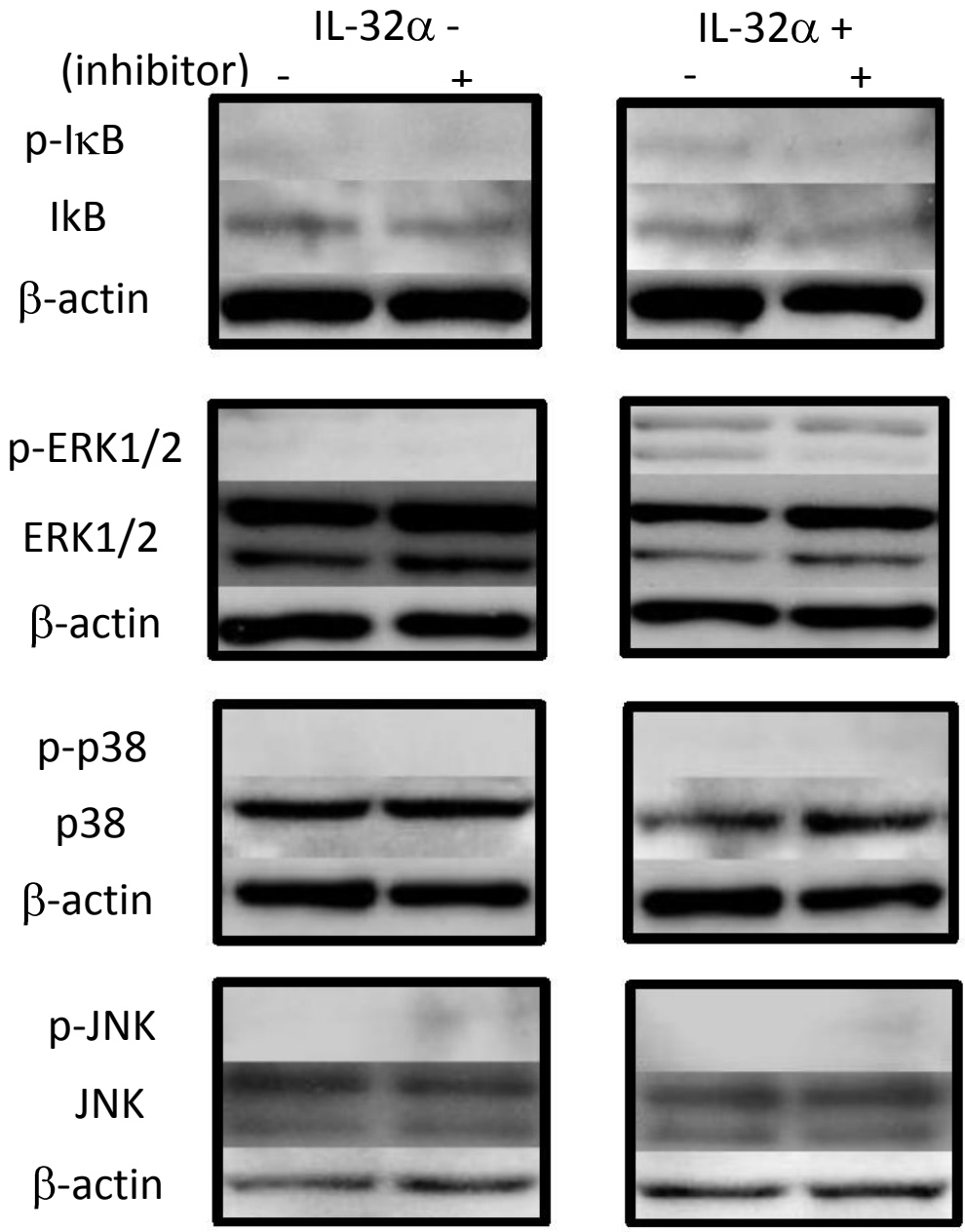

Supplement: Additional file 1 — Figure S1. Phosphorylation of IκB and MAPKs stimulated with rIL-32α (100 ng/ml) in RAW 264.7 cells was determined by Western blotting using anti-phospho-IκB, -ERK1/2, -p38, and -JNK antibodies. Phosphorylation of IκB and ERK1/2 were observed and inhibited by their specific inhibitors, while significant phosphorylation of p38 or JNK. was not observed. This data represents one of three independent experiments. [file ar3850-S1.PDF]

Figure S2

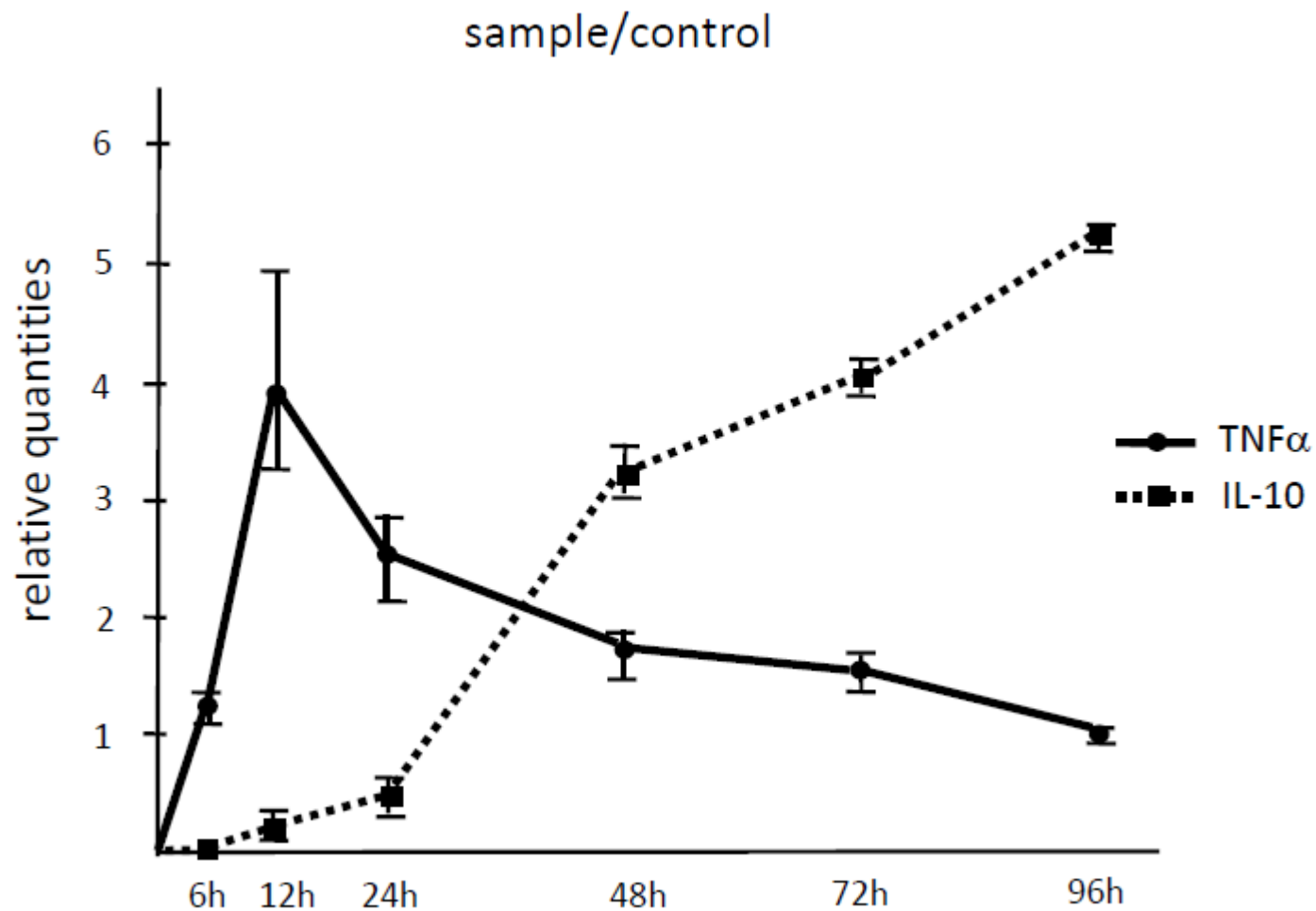

Supplement: Additional file 2 — Figure S2. Levels of TNFα and IL-10 were expressed as a proportion to that in control culture media without IL-32α stimulation. Level of TNFαpeaked at 12 h after stimulation with IL-32α and gradually decreased thereafter, while levels IL-10 kept increasing from 24 to 96 h after stimulation. Values are expressed as mean ± SD of triplicate determinations. [file ar3850-S2.PDF]
